# The optimal chemotherapeutic regimen in D2-resected locally advanced gastric cancer: a propensity score-matched analysis

**DOI:** 10.18632/oncotarget.16301

**Published:** 2017-03-16

**Authors:** Jun Eul Hwang, Myung Seo Ki, Karham Kim, Sung-Hoon Jung, Hyun-Jeong Shim, Woo-Kyun Bae, Eu-Chang Hwang, Young Hoe Hur, Oh Jeong, Seong Yeob Ryu, Young Kyu Park, Sang-Hee Cho, Ju-Seog Lee, Ik-Joo Chung

**Affiliations:** ^1^ Department of Hematology-Oncology, Chonnam National University Hwasun Hospital, Jeonnam, Korea; ^2^ Department of Urology, Chonnam National University Hwasun Hospital, Jeonnam, Korea; ^3^ Department of General Surgery, Chonnam National University Hwasun Hospital, Jeonnam, Korea; ^4^ Chonnam National University Hwasun Hospital, Jeonnam, Korea; ^5^ Department of Systems Biology, University of Texas MD Anderson Cancer Center, Houston, TX, USA

**Keywords:** gastric cancer, gastrectomy, prognosis, platinum, adjuvant chemotherapy

## Abstract

Adjuvant chemotherapy using TS-1 or capecitabine plus oxaliplatin improves survival outcomes after radical gastrectomy, with both regimens showing similar efficacies.

A total of 494 patients with stage II‒III gastric cancer who underwent curative D2 gastrectomy and received adjuvant chemotherapy from April 2004 to June 2014 were included in this study. 219 patients received TS-1, and 275 received platinum-based chemotherapy. The disease-free survival associated with adjuvant chemotherapy with TS-1 was compared with that associated with fluoropyrimidine plus platinum chemotherapy to identify the subgroups that would benefit most from platinum-based chemotherapy. The platinum group consisted of younger individuals, more males and more stage III patients compared with the TS-1 group. To reduce selection bias and its effects on treatment results, we performed a propensity score-matched analysis.

The matched cohort consisted of 219 TS-1 and 219 platinum treatment patients, respectively. In the matched cohort, the chemotherapeutic regimen did not affect disease-free survival according to stage (stage II: platinum vs. TS-1, *P* = 0.348; stage III: *P* = 0.132).

According to the subgroup analysis, platinum-based chemotherapy resulted in an improved 3-year disease-free survival compared with TS-1 treatment (66.8% vs. 57.8%, *P* = 0.015) for patients with high-risk features (any two or more of pT4, pN3, and lymphovascular invasion positivity).

Our results suggest that TS-1 alone is acceptable for patients without high-risk features, while platinum-based adjuvant chemotherapy should be administered to patients with high-risk features in D2-resected gastric cancer.

## INTRODUCTION

Gastric cancer is the third leading cause of cancer-related mortality worldwide, and in Korea, despite its decreasing incidence and mortality, gastric cancer remains the most common cancer in men and the third most frequent cause of cancer death in both sexes [1-3].

The only curative treatment for gastric cancer is surgery. Recent trials (CLASSIC and the Japanese ACTS-GC trial) demonstrated that adjuvant chemotherapy involving TS-1 or capecitabine plus oxaliplatin (CAPOX) after gastrectomy with D2 lymph node dissection resulted in improved disease-free survival (DFS) and overall survival (OS) compared with surgery alone in patients with stage II or III gastric cancer [4-8]. These two adjuvant chemotherapy regimens also demonstrated similar efficacies. The 3-year and 5-year OS rates were 80.1% and 71.7%, respectively, in the TS-1 trial and the 3-year DFS and 5-year OS rates were 74% and 78%, respectively, in the CAPOX trial. According to the subgroup analysis, CAPOX resulted in a significantly improved OS rate based on an increase in nodal status (N0: hazard ratio [HR] 0.79, 95% confidence interval [CI] 0.32‒1.95; N1 or N2, HR 0.67, 95% CI 0.51‒0.87). However, the efficacy of TS-1 was somewhat decreased in patients with N2 nodal status (N0: HR 0.317, 95% CI 0.127‒0.790; N1: HR 0.608, 95% CI 0.440‒0.840; N2: HR 0.839, 95% CI 0.612‒1.1150) [5, 7].

In this single-center study, we analyzed the efficacy of TS-1 and platinum-based adjuvant chemotherapy regimens (platinum group: CAPOX and 5-fluorouracil [5-FU] plus cisplatin [FP]) in patients with D2-resected stage II or III gastric cancer to identify the subgroup that would benefit most from TS-1 or platinum-based chemotherapy.

## RESULTS

Descriptive statistics related to the patient, tumor, and treatment characteristics for the entire cohort (n = 494), as well as the propensity score-matched cohort (n = 438), are summarized in Table 1 and 2.

**Table 1:** Baseline characteristics of the patients in the entire cohort (n = 494) stratified by adjuvant chemotherapy (platinum group vs. TS-1 group).

	Entire cohort		
Variable	Platinum group (n=275)	TS-1 (n=219)	*P*
Age, years			0.002
< 59, n. (%)	151 (54.9)	89 (40.6)	
≥59, n.(%)	124 (45.1)	130 (59.4)	
Regimen			
CAPOX	54 (19.6)	TS-1 (100)	
FP	221 (80.4)		
Sex			0.030
Male, n. (%)	202 (73.5)	141 (64.4)	
Female, n. (%)	73 (26.5)	78 (35.6)	
Tumor location			0.901
GEJ, whole stomach	59 (21.5)	48 (21.9)	
body, antrum	216 (78.5)	171 (78.1)	
Tumor grade			0.490
well/moderately differentiated	80 (29.1)	70 (32)	
poorly/un-differentiated	195 (70.9)	149 (68)	
Lauren classification			0.832
intestinal	137 (49.8)	107 (48.9)	
non-intestinal (diffuse or mixed)	138 (50.2)	112 (51.1)	
AJCC stage			0.003
II	95 (34.5)	105 (47.9)	
IIA/IIB	35 (12.7)/60 (21.8)	47 (21.5)/58 (26.5)	
III	180 (65.5)	114 (52.1)	
IIIA/IIIB/IIIC	63 (22.9)/69 (25.1)/48(17.5)	42 (19.2)/33 (15.1)/39 (17.8)	
T stage			0.354
T1	9 (3.3)	10 (4.6)	
T2	37 (13.5)	33 (15.1)	
T3	102 (37.1)	92 (42)	
T4	127 (46.2)	84 (38.4)	
N stage			0.309
N0	35 (12.7)	39 (17.8)	
N1	66 (24.0)	57 (26.0)	
N2	81 (29.5)	61 (27.9)	
N3	93 (33.8)	62 (28.3)	
LVI +	167 (60.7)	122 (55.7)	0.261
PNI+	198 (72.0)	160 (73.1)	0.793

5-*FU* 5-fluorouracil, *GEJ* gastroesophageal junction, *LVI* lymphovascular invasion, *PNI* perineural invasion

All patients median age (interquartile range, IQR): 59 (49-69)

**Table 2:** Baseline characteristics of the patients in the propensity score-matched cohort (n = 438) stratified by adjuvant chemotherapy (platinum group vs. TS-1 group).

	Propensity score-matched cohort		
Variable	Platinum group (n=219)	TS-1 (n=219)	*P*
Age, years			0.628
< 59, n. (%)	95 (43.4)	89 (40.6)	
≥59, n.(%)	124 (56.6)	130 (59.4)	
Regimen			
CAPOX	43 (19.6)	TS-1 (100)	
FP	176 (80.4)		
Sex			0.152
Male, n. (%)	156 (71.2)	141 (64.4)	
Female, n. (%)	63 (28.8)	78 (35.6)	
Tumor location			
GEJ, whole stomach	43 (19.6)	48 (21.9)	0.638
body, antrum	176 (80.4)	171 (78.1)	
Tumor grade			0.838
well/moderately differentiated	72 (32.9)	70 (32)	
poorly/un-differentiated	147 (67.1)	149 (68)	
Lauren classification			
intestinal	119 (54.3)	107 (48.9)	0.293
non-intestinal (diffuse or mixed)	100 (45.7)	112 (51.1)	
AJCC stage			0.388
II	95 (43.4)	105 (47.9)	
IIA/IIB	35 (16.0)/60 (27.4)	47 (21.5)/58 (26.5)	
III	124 (56.6)	114(52.1)	
IIIA/IIIB/IIIC	47 (21.5)/49 (22.4)/28 (12.8)	42 (19.2)/33 (15.1)/39 (17.8)	
T stage			0.851
T1	9 ( 4.1%)	10 ( 4.6%)	
T2	34 (15.5%)	33 (15.1%)	
T3	84 (38.4%)	92 (42.0%)	
T4	92 (42.0%)	84 (38.4%)	
N stage			0.915
N0	34 (15.5%)	39 (17.8%)	
N1	61 (27.9%)	57 (26.0%)	
N2	63 (28.8%)	61 (27.9%)	
N3	61 (27.9%)	62 (28.3%)	
LVI +	125 (57.1%)	122 (55.7%)	0.847
PNI+	147 (67.1%)	160 (73.1%)	0.210

5-*FU* 5-fluorouracil, *GEJ* gastroesophageal junction, *LVI* lymphovascular invasion, *PNI* perineural invasion

All patients median age (interquartile range, IQR): 59 (49-69)

### Entire cohort

Among the entire cohort, the platinum group, compared with the TS-1 group, comprised younger patients (age < 59 years: 54.9% *vs*. 40.6%), more males (73.5% *vs*. 64.4%), and more stage III patients (65.5% *vs*. 52.1%). A total of 275 patients received the platinum-based regimen, of whom 54 (19.6%) patients received CAPOX and 221 (80.4%) FP. All patients in the TS-1 group received TS-1. The median numbers of delivered cycles of FP, CAPOX, and TS-1 chemotherapy were 6 (range 1‒6, mean 5.37 ± 1.372), 8 (range 1‒8, mean 7.137 ± 2.210), and 8 (range 1‒8, mean 6.927 ± 2.340), respectively. The two groups were similar in terms of tumor location, tumor grade, Lauren classification, pT stage, pN stage, lymphovascular invasion (LVI), and perineural invasion.

The median follow-up period was 53.9 months (range 1.5‒119.9 months) in the platinum group and 48.2 months (range 2.4‒95.8 months) in the TS-1 group. In total, 164 events occurred: 90 (18.2%) in the platinum group and 74 (15%) in the TS-1 group. Distinct DFS curves were apparent according to stage (*P* < 0.0001, IIA‒IIIB: median DFS, not reached; IIIC: median DFS 23.3 months, 95% CI 10.872‒35.728). There was no statistically significant difference in DFS between the platinum and TS-1 groups in the entire cohort (*P* = 0.506). In the subgroup analysis, there were no differences in survival between the two groups according to stage (stage II: platinum *vs*. TS-1, *P* = 0.348; stage III: *P* = 0.190), pT stage, pN stage, or LVI status.

### Propensity score-matched cohort

Propensity score matching resulted in 219 matched pairs for a total of 438 patients. None of the clinical variables were significantly different between groups of matched pairs, indicating that the matching procedure was successful (Table 2).

**Figure 1:**
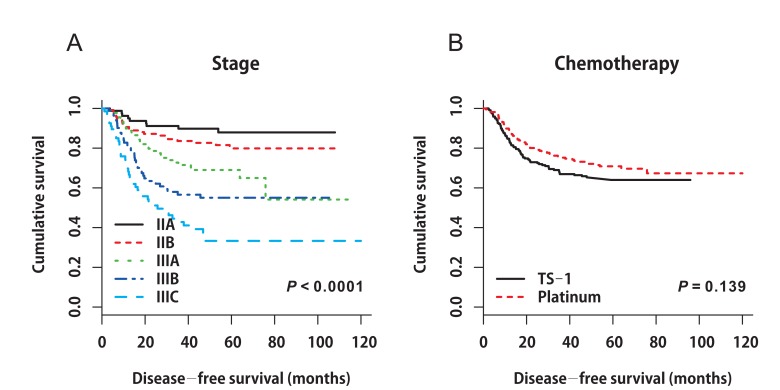
**A.** Disease-free survival curves stratified by stage in the propensity score-matched cohort (*P* < 0.0001, IIA‒IIIB: median DFS not reached; IIIC: median DFS 25.767 months, 95% CI 10.190‒41.344). **B.** Disease-free survival curves stratified by adjuvant chemotherapy in the propensity score-matched cohort (*P* = 0.139).

**Figure 2:**
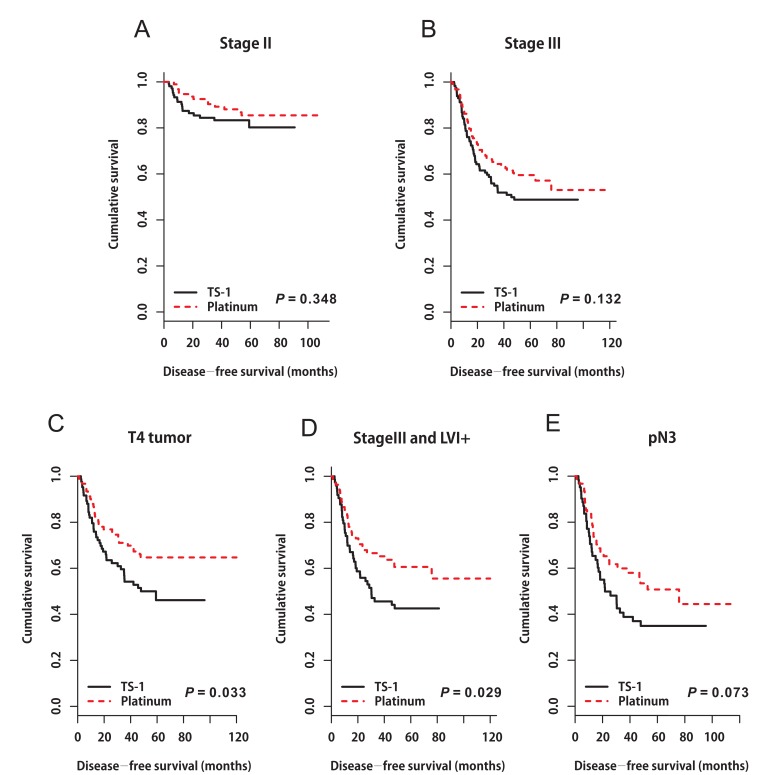
Disease-free survival curves stratified by adjuvant chemotherapy in patients with A stage II tumors (*P* = 0.348), **B.** stage III tumors (*P* = 0.132), **C.** pT4 tumors (*P* = 0.033), **D.** combined stage III and LVI+ tumors (*P* = 0.029), and **E**. pN3 nodal status (*P* = 0.073).

In the matched cohort, the median follow-up period was 55.0 months (range 1.5‒119.9 months) in the platinum group and 48.2 months (range 2.4‒95.8 months) in the TS-1 group. In total, 137 events occurred: 63 (14.4%) in the platinum group and 74 (16.9%) in the TS-1 group. Distinct DFS curves were apparent according to stage (Figure 1A, *P* < 0.0001, IIA‒IIIB: median DFS not reached; IIIC: median DFS 25.767 months, 95% CI 10.190‒41.344). The DFS curves did not differ between the platinum and TS-1 groups in the propensity score-matched population. The 3-year DFS rate was 84.8% in the platinum group and 66.9% in the TS-1 group (Figure 1B, *P* = 0.139). In the multivariate analysis, advanced T stage (pT3+pT4), advanced nodal status (pN2+pN3), and LVI positivity (LVI+) were identified as poor prognostic clinical variables for DFS (Table 3). In the subgroup analysis, there was no difference in survival between the two groups according to stage (Figure 2A, 2B, stage II: platinum *vs*. TS-1, p=0.348; stage III: platinum *vs*. TS-1, *P* = 0.132, median DFS, not reached *vs*. 45.63 months). However, platinum-based chemotherapy resulted in improved DFS in patients with pT4 tumors (Figure 2C, *P* = 0.033, platinum *vs*. TS-1, median DFS, not reached *vs*. 47.83 months) and in patients with combined stage III and LVI+ tumors (Figure 2D, *P* = 0.029, platinum vs. TS-1, median DFS, not reached *vs*. 30.267 months, 95% CI 6.650‒53.883). In patients with pN3 nodal status, platinum-based chemotherapy resulted in marginally improved DFS compared with TS-1 (Figure 2E, *P* = 0.073, platinum *vs*. TS-1, median DFS, 75.8 months, 95% CI 33.551‒118.049, *vs*. 21.767 months, 95% CI 7.367‒36.166).

**Table 3:** Univariate and multivariate analyses of risk factors for disease-free survival in the propensity score-matched cohort (n = 438).

Variables	Univariate analysis		Multivariate analysis	
HR (95% CI)	*P*	HR (95% CI)	*P*
Age ≥59, n.(%)	1.553 (1.094-2.205)	0.014	1.415 (0.979-2.044)	0.064
Male, n. (%)	1.168 (0.812-1.680)	0.403	1.476 (1.010-2.157)	0.044
Tumor location GEJ, whole stomach	1.133 (0.756-1.699)	0.545		
Lauren classification non-intestinal (diffuse or mixed)	1.252 (0.895-1.753)	0.189		
TS-1	1.289 (0.920-1.804)	0.14	1.288 (0.915-1.811)	0.146
T3+T4	2.704 (1.526-4.789)	0.001	2.799 (1.531-5.116)	0.001
N2+N3	3.188 (2.135-4.760)	0.0001	2.999 (1.981-4.540)	0.0001
LVI+	1.697 (1.190-2.420)	0.003	1.581 (1.089-2.296)	0.016
PNI+	1.435 (0.970-2.123)	0.071		

*GEJ* gastroesophageal junction

**Figure 3:**
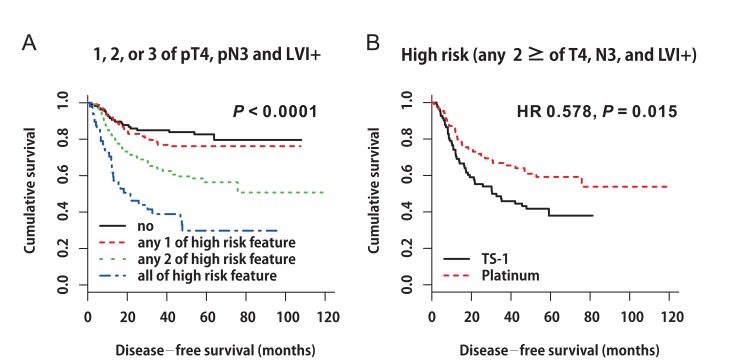
**A.** Disease-free survival curves according to the existence of pT4, pN3, and LVI+ (*P* < 0.0001). **B.** Disease-free survival curves stratified by adjuvant chemotherapy in patients with high-risk features (two or more of pT4, pN3, and LVI+) (HR 0.578, *P* = 0.015).

**Figure 4:**
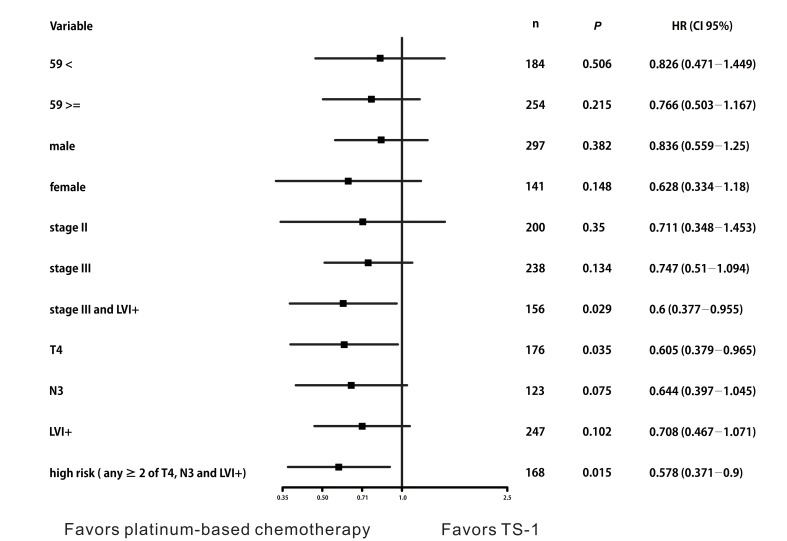
Disease-free survival stratified by adjuvant chemotherapy High-risk patients who benefited most from platinum-based chemotherapy (HR 0.578, 95% CI 0.371‒0.9, *P* = 0.015).

Based on these findings, we constructed DFS curves according to the existence of pT4, pN3, and LVI+ (Figure 3A, *P* < 0.0001). We found that patients with high-risk features (two or more of pT4, pN3, and LVI+) had better DFS when they received platinum-based chemotherapy (Figure 3B, platinum *vs*. TS-1, median DFS: not reached *vs*. 30.4 months, 95% CI 9.499‒51.301, *P* = 0.015). The 3-year DFS rate of patients with high-risk features was 66.8% in the platinum group and 57.8% in the TS-1 group. High-risk patients benefited the most from platinum-based chemotherapy (Figure 4, HR 0.578, 95% CI 0.371‒0.9, *P* = 0.015).

## DISCUSSION

Gastric cancer disseminates early via the lymphatic system, blood, and peritoneum. Recurrences or metastasis after surgery are common, and if they occur, the disease has a very dismal prognosis [9, 10]. Several attempts to reduce tumor recurrence have been made over the past decades using perioperative chemotherapy, postoperative chemoradiation, and chemotherapy [11-14]. Two recent adjuvant chemotherapy trials (CLASSIC and the Japanese ACTS-GC trial) of CAPOX and TS-1 demonstrated improved DFS and OS in patients with stage II or III gastric cancer who underwent D2 gastrectomy compared with those treated with surgery alone, and these results showed similar efficacies. However, until now, it was unknown as to which subgroup would benefit most from CAPOX or TS-1 (i.e., who would benefit more from adding the platinum compound to fluoropyrimidines). In this study, we demonstrated an increased DFS after adjuvant chemotherapy with a platinum-based regimen, compared with adjuvant TS-1 chemotherapy, in patients with high-risk features (two or more of pT4, pN3, and LVI+) who underwent D2 gastrectomy.

In colon cancer, adding oxaliplatin to 5-FU and leucovorin (i.e., the FOLFOX regimen) is recommend for patients with stage III and high-risk stage II colon cancer (high-risk features: poorly differentiated histology, LVI, perineural invasion, bowel obstruction, and localized perforation) [15-17]. Similarly, in this study, we demonstrated that platinum-based chemotherapy resulted in improved DFS in patients with combined stage III and LVI+ tumors. (Figure 2D, *P* = 0.029, Platinum-based chemotherapy *vs*. TS-1, median DFS, not reached *vs*. 30.267 months, 95% CI 6.650‒53.883).

A recent study reported that clinicians prefer CAPOX for younger patients and those with stage III gastric cancer; however, for elderly patients, clinicians choose TS-1 more often [18]. Similar to these findings, in this study, the platinum group, compared with the TS-1 group, was younger (age < 59 years: 54.9% *vs*. 40.6%) and included more stage III patients (65.5% *vs*. 52.1%). Generally, clinicians are reluctant to administer platinum to early-stage disease and elderly patients.

Doublet chemotherapy with fluoropyrimidine (5-FU or capecitabine) and platinum (cisplatin or oxaliplatin) is recommended for metastatic or locally advanced gastric cancer if the tumor is negative for HER2 amplification. In these cases, TS-1 and cisplatin, compared with TS-1 alone, resulted in improved progression-free survival (TS-1/cisplatin *vs*. TS-1: 6.0 *vs*. 4.0 months, *P* < 0.0001) and OS (13.0 *vs*. 11.0 months, *P* = 0.04) [19-21]. Similar to metastatic gastric cancer, platinum-based adjuvant chemotherapy can be a better option for advanced-stage and high-risk patients even in the adjuvant setting, as shown here in D2-resected gastric cancer patients with high-risk features treated with platinum-based chemotherapy.

There are several limitations to this study. This was a retrospective analysis from a single institution. We did not present the toxicities associated with each chemotherapy regimen; however, all regimens are widely used in the clinical setting, and all toxicities were manageable and did not differ from those reported in previous studies. Therefore, there were no special toxicities to report. Additionally, we did not evaluate OS. Considering the retrospective nature of this adjuvant chemotherapy study, various confounding factors for OS may exist, such as loss to follow-up, the general medical conditions of the patient, and palliative chemotherapy (whether the patient received it or not after the disease recurred). According to a previous report, in patients with gastric cancer, most relapses occur within 3 years of surgery, and DFS is an acceptable surrogate endpoint in place of OS in trials of adjuvant chemotherapy for gastric cancer [22, 23]. Therefore, investigation of DFS was justified in this adjuvant chemotherapy study.

In conclusion, TS-1 alone is acceptable for D2-resected gastric cancer patients without high-risk features, while platinum-based adjuvant chemotherapy should be considered for patients with high-risk features, especially stage III patients. These findings should be further confirmed in a randomized prospective clinical trial.

## MATERIALS AND METHODS

### Patients

A total of 494 patients with gastric cancer received adjuvant chemotherapy after D2 gastrectomy between April 2005 and June 2014 at the Chonnam National University Hwasun Hospital in Jeonnam, Republic of Korea. Patients with no adjuvant chemotherapy after surgery for any reason (n = 137), other co-existing cancers (n = 9), palliative resection (n = 77), and pathologic evidence of distant metastasis (n = 40) were excluded. The inclusion criteria were as follows: histologically confirmed stage II or III gastric adenocarcinoma according to the American Joint Committee on Cancer, 7^th^ edition, no evidence of distant metastasis, R0 resection (with no tumor cells in the surgical margin), D2 lymph node dissection, and no previous cancer treatment. All data were collected from our institutional data base, and the survival data were updated at the time of analysis. This study was approved by the Institutional Review Board of Chonnam National University Medical School Research Institution, which waived the requirement for written informed consent from the patients due to the retrospective nature of this study.

### Adjuvant chemotherapy

Adjuvant chemotherapy was administered using TS-1 (Taiho Pharmaceutical, Tokyo, Japan), FP, or CAPOX according to the physician’s judgment and patient’s preference. The FP regimen was used most commonly, because the Korea National Health Insurance Service did not reimburse for the CAPOX regimen prior to March 2013. Since April 2013, CAPOX has been used instead of FP due to insurance reimbursement from the Korea National Health Insurance Service. The dose of TS-1 was determined based on the body surface area (BSA). Accordingly, patients received one of the following doses, divided into two, after meals daily: 80 mg for patients with a BSA < 1.25 m^2^, 100 mg for those with a BSA of 1.25‒1.49 m^2^, and 120 mg for those with a BSA ≥ 1.50 m^2^. TS-1 was administered for 4 weeks followed by a 2-week break. Treatment was continued for 1 year after surgery. The FP regimen was as follows: 5-FU (800 mg/m^2^ per day) was administered by continuous intravenous infusion on days 1‒5 of each cycle, and cisplatin (80 mg/m^2^) was administered by intravenous infusion on day 1. The FP regimen was administered every 4 weeks for 6 cycles. The CAPOX regimen was administered over eight 3-week cycles, consisting of capecitabine (1000 mg/m^2^ twice daily on days 1‒14 of each cycle) plus intravenous oxaliplatin (130 mg/m^2^ on day 1 of each cycle). The management of adverse events and subsequent dose reductions of the chemotherapeutic agents were performed according to a conventional protocol.

### Follow-up

To assess tumor recurrence, abdominal computed tomography was performed every 3 months during the first 2 years after surgery and every 6 months thereafter until 5 years after surgery. Physical examination, chest radiography, and measurements of carcinoembryonic antigen and carbohydrate antigen 19-9 tumor markers were performed every 3 months for the first 2 years and every 6 months thereafter until 5 years. If clinical signs or symptoms indicated possible recurrence or the development of a new gastric cancer, an investigation was conducted to verify whether the patient was disease-free.

### Statistical analysis

Statistically significant differences were assessed using the Chi squared test or Fisher’s exact test for categorical data. The t-test or Mann-Whitney U test was used for continuous data. Since patients were not randomly assigned to receive postoperative adjuvant TS-1 or platinum-based chemotherapy (platinum group: FP or CAPOX), it was highly likely that the two patient groups would have significant baseline differences that could confound the final outcome analysis. To reduce the effects of selection bias and potential confounding factors, such as uneven patient distribution between the TS-1 and platinum group, we used 1:1 propensity score matching to adjust for age, sex, and stage.

DFS was defined as the time from the date of surgery to the detection of recurrent disease or death, whichever occurred first. If neither event occurred at the time of analysis, the patient was censored. Survival curves were generated using the Kaplan-Meier method, and a survival comparison was performed using the log-rank test. Factors associated with DFS were determined by univariate and multivariate Cox proportional hazard regression models with hazard ratios (HRs) and 95% confidence intervals (CIs). All statistical analyses were performed using SPSS version 21.0 (IBM Corporation, Armonk, NY, USA) and the program R (R Foundation for Statistical Computing, Vienna, Austria, http://www.R-project.org). All p values are two-sided, with p < 0.05 considered statistically significant.
